# Effects of Stereotactic Body Radiation Therapy Plus PD-1 Inhibitors for Patients With Transarterial Chemoembolization Refractory

**DOI:** 10.3389/fonc.2022.839605

**Published:** 2022-03-21

**Authors:** Yan-Jun Xiang, Kang Wang, Yi-Tao Zheng, Shuang Feng, Hong-Ming Yu, Xiao-Wei Li, Xi Cheng, Yu-Qiang Cheng, Jin-Kai Feng, Li-Ping Zhou, Yan Meng, Jian Zhai, Yun-Feng Shan, Shu-Qun Cheng

**Affiliations:** ^1^Department of Hepatic Surgery VI, Eastern Hepatobiliary Surgery Hospital, Naval Medical University, Shanghai, China; ^2^Department of Hepatobiliary Surgery, The First Affiliated Hospital, Wenzhou Medical University, Wenzhou, China; ^3^Department of Radiotherapy, Eastern Hepatobiliary Surgery Hospital, Naval Medical University, Shanghai, China; ^4^Department II of Interventional Radiology, Eastern Hepatobiliary Surgery Hospital, Naval Medical University, Shanghai, China

**Keywords:** hepatocellular carcinoma, stereotactic body radiation therapy, transarterial chemoembolization refractory, immunotherapy, combination therapy

## Abstract

**Background and Aims:**

Patients with intermediate-stage hepatocellular carcinoma (HCC) who are refractory to transarterial chemoembolization (TACE) have a poor prognosis. This study aimed to explore whether stereotactic body radiation therapy (SBRT) combined with PD-1 inhibitors could improve the clinical outcomes of such patients.

**Methods:**

This retrospective cohort study included patients with intermediate-stage HCC who were diagnosed with TACE refractoriness between January 2019 and December 2020 in the Eastern Hepatobiliary Surgery Hospital and the First Affiliated Hospital of Wenzhou Medical University. The patients were divided into two groups: (1) those who switched from TACE to receive stereotactic body radiotherapy (SBRT) combined with PD-1 inhibitors; (2) those who continued TACE treatment and added PD-1 inhibitors. Progression-free survival (PFS), overall survival (OS), and tumour response were assessed in both groups after becoming refractory to TACE treatment.

**Results:**

Of the seventy-six patients included in this study, the median PFS was 19.6 months in the SBRT-IO group (n=31) and 10.1 months in the TACE-IO group (n=45, p<0.05). The SBRT-IO group also had a significantly higher OS than the TACE-IO group (p<0.05). The objective response rate (ORR) and disease control rate (DCR) were also better in the SBRT-IO group (ORR, 71.0% vs. 15.6%, OR=8.483, 95% CI 3.319-21.680, P < 0.001; DCR, 80.6% vs. 31.1%, OR=9.226, 95% CI 3.096-27.493, P < 0.001).

**Conclusions:**

SBRT combined with a PD-1 inhibitor improves PFS and OS in TACE-refractory patients with intermediate-stage HCC. Therefore, this therapy is a suitable option in cases of TACE treatment failure.

## Introduction

Hepatocellular carcinoma (HCC) is the most common primary liver cancer and the fourth leading cause of cancer-related death worldwide (1). Since patients with early-stage HCC are usually asymptomatic, approximately half of them are diagnosed at intermediate to advanced stages and cannot undergo radical treatment (2–5).

For patients with intermediate-stage HCC, transarterial chemoembolization (TACE) is recommended as the standard treatment by many guidelines (6–9). However, the efficacy of TACE alone is limited, and some patients are diagnosed as refractory to TACE (10, 11). Most guidelines recommend starting systemic therapy as soon as TACE refractoriness occurs (6, 8, 9). As a new systemic therapeutic drug, PD-1 inhibitors show synergistic effects when combined with TACE (12, 13). In other words, the combined use of PD-1 inhibitors may improve the prognosis of TACE-refractory patients.

Stereotactic body radiotherapy (SBRT) is a newer treatment with evidence of promising local control for patients with HCC (14–16). For early- and intermediate-stage HCC patients, SBRT is a safe alternative to TACE and provides no inferior or even better local control and overall survival (OS) than TACE (17, 18). Furthermore, there is synergy in the use of radiotherapy in combination with PD-1 inhibitors (19, 20). Therefore, we speculate that SBRT combined with a PD-1 inhibitor may be an effective alternative treatment for TACE-refractory patients.

In this study, we investigated whether TACE-refractory patients should be administered PD-1 inhibitors to maintain TACE treatment or should be switched to SBRT plus PD-1 inhibitors, as reports on these two treatments are currently lacking. We conducted this retrospective study to evaluate the efficacy and safety of the above two therapies in intermediate HCC patients who are refractory to TACE treatment.

## Methods

### Patients

A retrospective study of consecutive HCC patients was conducted at the Eastern Hepatobiliary Surgery Hospital and the First Affiliated Hospital of Wenzhou Medical University from 2019 to 2020. This study was approved by the Institutional Ethics Committee of each centre. As patient identities were anonymized, the requirement for informed consent was waived by the ethics committee.

The inclusion criteria were patients with (1) HCC diagnosed by histopathology, computed tomography (CT) or magnetic resonance imaging (MRI), (2) good liver function (Child-Pugh A or B7, score <= 7), (3) BCLC stage B, (4) TACE, and (5) TACE refractoriness. The exclusion criteria were patients with (1) previous locoregional or systemic therapy, (2) recurrent HCC, (3) a history of other cancers, and (4) incomplete clinical data.

### TACE and SBRT

The optimal treatment modality was discussed and determined by the multidisciplinary team at each institution. Locoregional therapies, including surgery or alternative approaches (SBRT or TACE), are considered based on the individual patient’s circumstances (tumour size, liver function, and proximity to organs at risk). The final decision is made by the patient after the benefits of various treatment modalities, as well as associated side effects and costs, have been fully explained.

TACE was performed as previously described using the Seldinger’s technique (21). Briefly, the tumour-feeding artery was first identified by angiography, and after cannulation of the hepatic artery, doxorubicin hydrochloride, pirarubicin and lipiodol were injected through the catheter. Post TACE evaluation and follow-up were performed every 6-8 weeks. The diagnostic criteria of TACE refractoriness were based on the definition proposed by the Japan Society of Hepatology (JSH) and the Liver Cancer Study Group of Japan (insufficient response of the treated tumour after two procedures) (22).

SBRT was performed by CyberKnife^®^ (Accuray Cyberknife, VSI), with a total of 24–45 Gy in 3–5 fractions. The patients who received SBRT were first implanted with at least 3 gold fiducials inside or adjacent to the tumour under CT (Philips Brilliance CT Big Bore Oncology) guidance, and the gold fiducials were relatively stable and immobile after seven days, with localization simulated under CT. The images were subsequently transferred to the treatment planning system, and the target area was then delineated by a radiologist. A 2-5 mm marginal expansion of the gross tumour volume (defined as radiologically evident gross disease) formed the planning target volume. The physiatrist developed the treatment plan while defining normal tissue dose ranges. Dose-volume histograms were generated for all target volumes and critical normal structures. Dose constraints for organs at risk were determined based on the American Association of Physicists in Medicine guidelines in AAPM Task Group 101 (23).

### PD-1 Inhibitors

All included patients were treated with PD-1 inhibitors after being diagnosed as refractory to TACE treatment. PD-1 inhibitors included toripalimab (72.4%) and sintilimab (27.6%) (**Supplementary Table 1**), both of which have been reported to be effective in patients with HCC (24–31). Toripalimab was administered at a dose of 3 mg/kg by body weight every 2 weeks; sintilimab was administered at a dose of 200 mg every three weeks. The specific doses and protocols used were strictly in accordance with the instructions for use. PD-1 inhibitors were all administered intravenously; if low-grade infusion reactions occurred, drip plasticity was reduced or dosing was suspended until the symptoms resolved, at which time the medication was resumed while the patient remained under close observation. PD-1 inhibitors were continued until intolerable toxicity occurred.

### Follow−Up and Assessment

All patients visited the outpatient clinic for follow-up every 1-3 months. At each follow-up visit, a routine physical examination, laboratory blood tests, and abdominal ultrasound or enhanced CT/MRI were performed. The primary outcome of this study was progression-free survival (PFS), which was defined as the time from the initiation of PD-1 inhibitors to tumour progression, death from any cause, or the most recent follow-up. The secondary endpoints included overall survival (OS), objective response rate (ORR) and treatment-related adverse events (TRAEs). Tumour progression included progression of treated lesion, and new lesions within or outside the liver. OS was defined as the time from the initiation of PD-1 inhibitor use until the date of death from any cause or the date of the most recent follow-up visit. Disease control rate (DCR) was defined as percentage of patient attained complete response, partial response or stable disease. Assessment of tumour progression was based on modified Response Evaluation Criteria in Solid Tumours criteria (mRECIST).

TRAEs were recorded from the initiation of PD-1 inhibitor use and obtained from clinical visit notes or medical records. TRAEs were assessed according to the criteria of the common terminology criteria for adverse events (CTCAE, version 5.0). If multiple instances of the same type of toxicity occurred, the highest grade for each patient in a given category was adopted.

### Statistical Analysis

All clinical data were analysed using IBM SPSS Statistics 24 (New York, NY, USA) or R 4.0 software (http://www.r-project.org/). Student’s t-test was used to compare continuous variables, and the χ2 test or Fisher exact test was used to compare categorical variables. Survival curves were calculated using the Kaplan–Meier method and compared using the log-rank test. The hazard ratio (HR) was calculated by Cox regression models. Univariate Cox regression analysis was used to evaluate the significance of variable in the entire cohort. All variables which were significantly related to PFS (p<0.05) were included in the multiple Cox regression analysis. The odds ratio (OR) was calculated by logistic regression models. P < 0.05 was considered to indicate a significant difference.

## Results

### Patient Characteristics and Treatments

**Figure 1:**
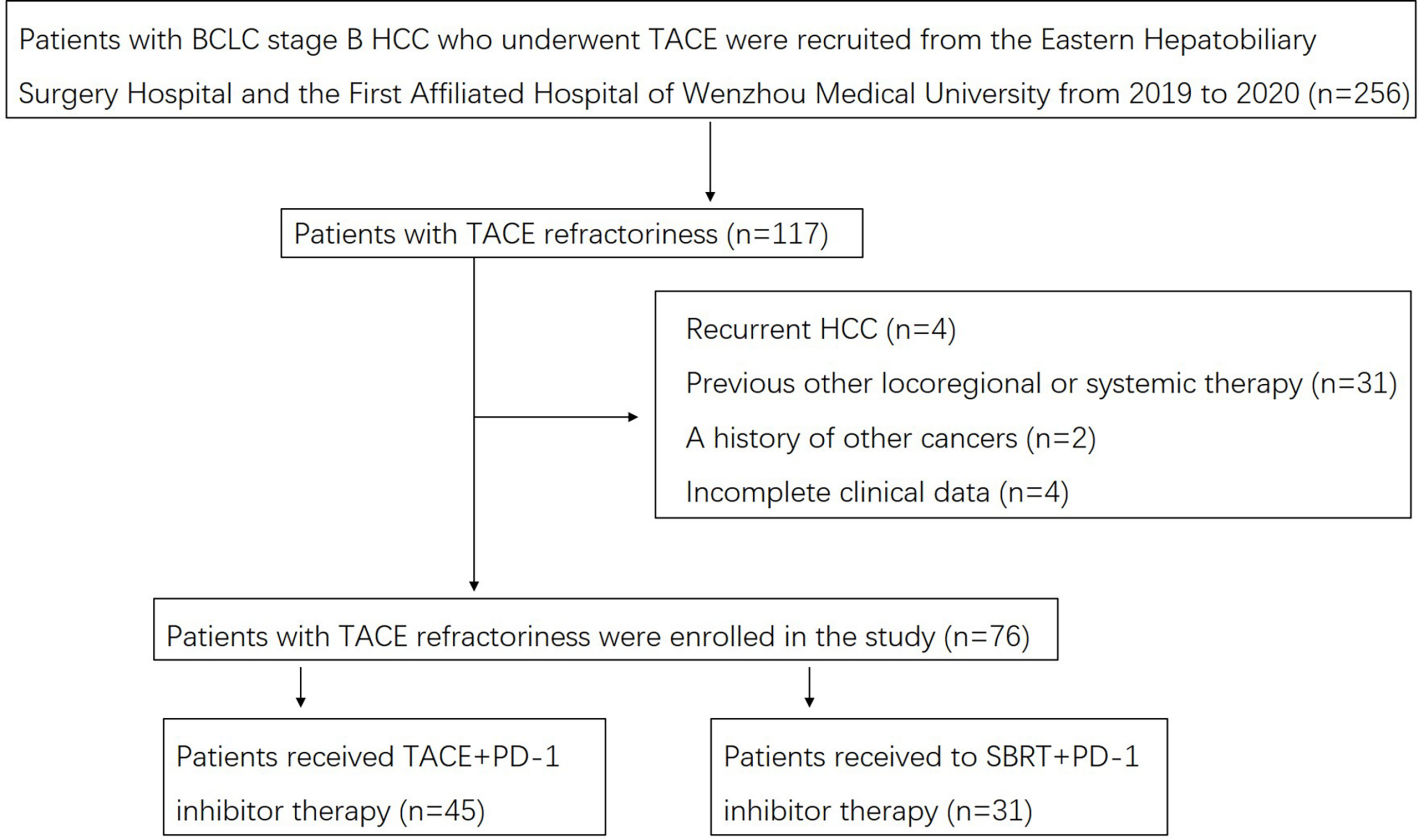
Flow diagram for the present study. BCLC, Barcelona Clinic Liver Cancer; HCC, hepatocellular carcinoma; TACE, transarterial chemoembolization; SBRT, stereotactic body radiation therapy.

A flow diagram of the present study is shown in **Figure 1**. Of the 76 patients at the Eastern Hepatobiliary Surgery Hospital and the First Affiliated Hospital of Wenzhou Medical University with complete clinical and follow-up data, 45 (59.2%) patients received TACE-IO therapy, and 31 (47.3%) received SBRT-IO therapy. **Table 1** summarizes the baseline features of these patients. There were no significant differences at baseline between the two groups, including age, sex, HBsAg, maximum tumour size, number of tumours, alpha-fetoprotein concentration, Des-gamma-carboxy prothrombin, total bilirubin, albumin, albumin-bilirubin grade, prothrombin time, glucose, creatinine or platelet count.

**Table 1:** Baseline characteristics of study patients.

Characteristics	TACE-IO (n = 45)	SBRT-IO (n = 31)	*P* value
Age, year			0.945
< 65	36 (86.2)	25 (78.8)	
≥ 65	9 (13.8)	6 (21.2)	
Gender			0.168
Female	6 (13.8)	8 (19.2)	
Male	39 (86.2)	23 (80.8)	
HBsAg			0.525
Positive	40 (82.8)	26 (78.9)	
Negative	5 (17.2)	5 (21.1)	
Liver cirrhosis			0.666
Yes	38 (84.4)	25 (80.6)	
No	7 (15.6)	6 (19.4)	
Child-Pugh			1.000
A	44	31	
B7	1	0	
Maximum tumour size, cm, median (range)	4.8 (1.3-12)	4.3 (1.6-6)	0.161
Tumour number			0.137
2	31	20	
3	10	11	
4	4	0	
AFP, ng/mL			0.610
< 400	22 (58.6)	17 (61.5)	
≥ 400	23 (41.4)	14 (38.5)	
DCP, mAU/mL			0.555
< 2050	26 (65.5)	20 (73.1)	
≥ 2050	19 (34.5)	11 (26.9)	
TB, umol/L			0.468
< 18.8	27	16	
≥ 18.1	18	15	
Albumin, g/L			0.145
< 35	8	10	
≥ 35	37	21	
ALBI grade			0.243
1	25 (55.2)	13 (53.9)	
2	20 (41.4)	18 (46.1)	
3	0	0	
PT, sec			0.669
< 13	36	26	
≥ 13	9	5	
Glucose, mmol/L			0.337
< 7	39	29	
≥ 7	6	2	
Creatinine, umol/L, median	66.0	61.0	0.222
Platelet, X10^9^, median	162.0	174.0	0.625

TACE, transcatheter arterial chemoembolization; SBRT, stereotactic body radiation therapy; AFP, alpha-fetoprotein concentration; DCP, Des-gamma-carboxy prothrombin; TB, total bilirubin; ALBI, albumin-bilirubin; PT, prothrombin time.

The median follow-up was 10 and 11 months in the TACE-IO and SBRT-IO groups, respectively. The median cycle of PD-1 inhibitor use was six in both groups. Total 63 lesions were irradiated in SBRT-IO arm (Single lesion, n=5; Two lesions, n=20; Three lesions, n=6). Of the 76 patients enrolled in the study, 41 patients died during the study (31 in the TACE-IO group and 10 in the SBRT-IO group), 29 were alive (10 in the TACE-IO group and 19 in the SBRT-IO group), and 6 were lost to follow-up (4 in the TACE-IO group and 2 in the SBRT-IO group).

### Efficacy Outcomes

**Figure 2:**
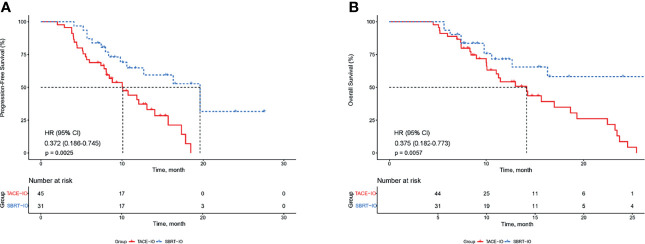
Kaplan–Meier estimated PFS and OS curves of HCC patients receiving different therapies. **(A)** PFS; **(B)** OS. PFS, progression-free survival; OS, overall survival; HCC, hepatocellular carcinoma; TACE, transarterial chemoembolization; SBRT, stereotactic body radiation therapy.

The median PFS was 19.6 months (95% CI 13.1-26.1) in the SBRT-IO group and 10.1 months (95% CI 7.3-12.9) in the TACE-IO group. The median OS was 14.1 months in the TACE-IO group and was not reached in the SBRT-IO group. The 1-year OS and PFS rates of the SBRT-IO group were 71.5% and 64.8%, respectively, while those of the TACE-IO group were 54.2% and 40.7%, respectively. SBRT significantly prolonged PFS relative to TACE (**Figure 2A**, P < 0.05). In the entire cohort, treatment with SBRT-IO was a significantly unfavourable factor for PFS (HR=0.372, 95% CI 0.186-0.745, P=0.005), along with ALBI grade 2 (**Table 2**). Similarly, as shown in **Figure 2B**, SBRT significantly prolonged OS relative to TACE (HR = 0.375, 95% CI 0.182-0.773, P < 0.05).

**Table 2:** Prognostic factors for progression-free survival.

Progression-free survival	Univariate analysis			Multivariate analysis		
	HR	95% CI	P value	HR	95% CI	P value
Age (>65/≤65 years)	1.459	0.669-3.183	0.342			
Sex (male/female)	2.029	0.857-4.808	0.108			
HBsAg (positive/negative)	0.614	0.283-1.334	0.218			
Albumin (>35/≤35 g/dl)	0.638	0.296-1.373	0.250			
Total bilirubin (>17.1/≤17.1 μmol/L)	1.524	0.838-2.773	0.167			
ALBI grade (2/1)	2.234	1.200-4.159	0.011	2.132	1.144-3.970	**0.017**
Prothrombin time (>13/≤13 second)	0.866	0.361-2.076	0.747			
Creatinine (>106/≤106 μmol/L)	1.001	0.982-1.021	0.896			
Blood glucose (>7/≤7 mmol/L)	1.031	0.401-2.649	0.950			
Platelet (>100/≤100 10^9/L)	0.995	0.988-1.003	0.216			
Alpha fetoprotein (≥400/<400 ng/mL)	0.770	0.427-1.391	0.386			
DCP (≥2050/<2050 mAU/mL)	0.730	0.396-1.346	0.314			
Liver cirrhosis (yes/no)	1.080	0.517-2.255	0.838			
Tumour number (3/2)	1.044	0.533-2.042	0.901			
Tumour number (4/2)	3.022	0.901-10.141	0.073			
Maximum tumour size, cm	1.126	0.575-2.205	0.730			
Treatment (SBRT-IO/TACE-IO)	0.361	0.182-0.716	0.004	0.372	0.186-0.745	**0.005**

ALBI, albumin-bilirubin; DCP, Des-gamma-carboxy prothrombin; SBRT, stereotactic body radiation therapy; TACE, transcatheter arterial chemoembolization.

**Table 3** summarizes the best tumour responses for all HCC patients. According to mRECIST, the ORR in the SBRT-IO group was significantly higher than that in the TACE-IO group (71.0% vs. 15.6%, OR=8.483, 95% CI 3.319-21.680, P < 0.001). The DCR in the SBRT-IO group was also significantly higher than that in the TACE-IO group (80.6% vs. 31.1%, OR=9.226, 95% CI 3.096-27.493, P < 0.001).

**Table 3:** Best tumour response.

	TACE-IO (n=45)	SBRT-IO (n=31)	*P* value
CR	0	11(35.5)	
PR	7 (15.6)	11 (35.5)	
SD	7 (15.6)	3 (9.7)	
PD	31 (68.9)	6 (19.4)	
ORR	7 (15.6)	22 (71.0)	<0.001
DCR	14 (31.1)	25 (80.6)	<0.001

TACE, transcatheter arterial chemoembolization; SBRT, stereotactic body radiation therapy; CR, complete response; PR, partial response; SD, stable disease; PD, progressive disease; ORR, objective response rate; DCR, disease control rate.

### First Site of Progressive Disease and Treatment on Progression

Forty-five patients had progressed at the time of analysis. Thirty-one (68.9%) patients in the TACE-IO cohort progressed, 26 (57.8%) of whom had intrahepatic progression; Fourteen (45.2%) patients progressed in the SBRT-IO cohort, 11 (35.5%) of whom had intrahepatic progression, as detailed in **Supplementary Table 2**.

Following progressive disease, most patients had more treatment. In the TACE-IO cohort, 27 patients received further treatment, three patients received supportive care due to physical deterioration, and one patient refused treatment; In the SBRT-IO cohort, 12 patients received further treatment, one patient received supportive care and one patient refused treatment.

### Safety Outcomes

According to CTCAE version 5.0, TRAEs were evaluated during treatment according to their frequency and severity. Almost all patients experienced transient TRAEs after receiving locoregional therapies, which spontaneously resolved. Therefore, we did not analyse and discuss these transient TRAEs.

As shown in **Supplementary Table 3**, the most common TRAEs at all levels in the TACE-IO group were decreased platelet count (44.4%), decreased albumin (37.8%), and elevated AST (37.8%). In addition, the most common grade 3/4 TRAE was decreased platelet count (6.7%). In the SBRT-IO group, the most common TRAEs were fatigue (54.8%), decreased platelet count (48.4%) and decreased white blood cell (32.3%), and the most common grade 3/4 TRAEs were elevated AST (3.2%) and ALT (3.2%) levels, and hand-foot skin reaction (3.2%). Furthermore, among patients treated with SBRT-IO, none developed classical radiation-induced liver disease, and no treatment-related deaths occurred.

## Discussion

In this study, we report for the first time the efficacy of SBRT combined with a PD-1 inhibitor in TACE-refractory patients with intermediate-stage HCC. The results showed that receiving SBRT combined with a PD-1 inhibitor provided a better long-term prognosis and greater tumour control than TACE combined with a PD-1 inhibitor alone for TACE refractory patients. This provides more options for the treatment of patients with BCLC stage B HCC.

TACE is the standard of care for patients with BCLC stage B HCC (6–9), but some patients develop TACE refractoriness and cannot achieve effective tumour control (10, 11). The guidelines recommend that patients start receiving systemic therapy once they are diagnosed with TACE refractoriness (6, 8, 9). However, a recent international expert panel of International Society of Multidisciplinary Interventional Oncology consensus statement and a survey by Chinese College of Interventionalists indicated that repeated TACE especially TACE based combination therapy can also achieve survival benefit in patients refractory to TACE (32–35). Meanwhile, PD-1 inhibitors have been increasingly explored as representative agents for immunotherapy, the possible mechanism underlying the benefit of TACE combined with a PD-1 inhibitor was revealed: TACE could decrease the ratio of CD4+/CD8+ cells and increase the level of PD-1 mRNA expression in patients with HCC (12). Therefore, TACE combined with a PD-1 inhibitor might have potential clinical value for patients who are refractory to TACE.

Radiotherapy is limited in its clinical application in these patients because of increased hepatotoxicity. Due to technological advances, SBRT is currently able to safely deliver high-dose radiotherapy to HCC patients, and the American Association for the Study of Liver Diseases guidelines accept SBRT as one of the treatments for HCC (7). A previous study showed that patients with intermediate- and advanced-stage HCC can also benefit from SBRT (36), and another study demonstrated that the 2-year local control rate reached 61-81% in patients with BCLC stage B HCC who received SBRT (37). Several retrospective controlled studies involving patients with intermediate-stage HCC showed that SBRT had similar or even higher tumour control rates and OS rates than TACE (17, 18), and one clinical trial demonstrated the safety and feasibility of SBRT as a local salvage regimen for patients with an incomplete response to TACE (38). On the one hand, the benefit of SBRT for patients with HCC is guaranteed, while on the other hand, the potential benefit of combining SBRT with a PD-1 inhibitor has been revealed. In terms of the underlying mechanism, radiotherapy can trigger immunogenic cell death, resulting in the release of cytokines and damage-associated molecular patterns (DAMPs). DAMPs can lead to the subsequent priming and trafficking of tumour-specific T lymphocytes into the tumour microenvironment by enhancing the recruitment of antigen-presenting cells, the processing of tumour-associated antigens, and the cross presentation of antigenic peptides on major histocompatibility complex class I, thereby enhancing the efficacy of PD-1 inhibitors (20). Its clinical benefits have also been reported (38–40).

Based on the above findings, we speculate that intermediate-stage HCC patients who are refractory to TACE might benefit from the addition of a PD-1 inhibitor or from the switch to SBRT combined with a PD-1 inhibitor. In this study, which enrolled 76 patients proven to be refractory to PD-1 inhibitor TACE treatment, the SBRT-IO group (n = 31) had a median PFS of 19.6 months (95% CI 13.1-26.1), which was significantly higher than the TACE-IO group (n = 45) with a median PFS of 10.1 months (95% CI 7.3-12.9, P < 0.001). The 1-year OS and 1-year PFS rates in the SBRT-IO group were 71.5% and 64.8%, and the ORR and DCR were 71.0% and 80.6%, respectively. The 1-year OS and 1-year PFS rates in the TACE-IO group were 54.2% and 40.7%, and the ORR and DCR were 15.6% and 31.1%, respectively. Compared with TACE-IO, SBRT-OI significantly prolonged PFS (HR=0.372, 95% CI 0.186-0.745, P=0.005) and OS (HR = 0.375, 95% CI 0.182-0.773, P < 0.001) and resulted in a better ORR (OR=8.483, 95% CI 3.319-21.680, P < 0.001) and DCR (OR = 9.226, 95% CI 3.096-27.493, P < 0.001) in TACE-refractory patients. Furthermore, the median OS of the TACE-IO group was similar to that of TACE-refractory patients as previously reported by Kudo et al. (41); therefore, whether adding a PD-1 inhibitor can improve the prognosis for TACE-refractory patients requires further study.

In addition to efficacy, we analysed the TRAEs associated with SBRT plus PD-1 inhibitors. The most common TRAEs were decreased WBC (67.7%), fatigue (54.8%) and decreased platelet count (48.4%), and the most common grade 3/4 TRAEs were decreased WBC (6.5%), elevated AST (3.2%) and ALT (3.2%) levels and hand-foot skin reaction (3.2%), with no unexpected TRAEs occurring. Therefore, SBRT-IO is an effective and safe treatment for intermediate-stage HCC patients who are refractory to TACE treatment.

We must acknowledge that our study had some limitations. First, this is a retrospective study with inherent defects. Second, this was a study conducted in HBV-endemic China, which may have influenced the results. Third, the sample size included in this study was small, and the number of tumours per patient was small (fewer than 5). A prospective study is therefore needed to confirm our findings.

In conclusion, our data strongly support the fact that switching to a combination of SBRT and a PD-1 inhibitor improves clinical outcomes, as evidenced by the increased PFS and OS in intermediate-stage HCC patients who are refractory to TACE. Repeated TACE treatments may cause resistance to systemic therapy and result in the deterioration of liver function. Therefore, the combination of SBRT with a PD-1 inhibitor is a safe and effective alternative that warrants consideration by clinicians.

## Data Availability Statement

The raw data supporting the conclusions of this article will be made available by the authors, without undue reservation.

## Ethics Statement

This study was approved by the Institutional Ethics Committees of the Eastern Hepatobiliary Surgery Hospital and the First Affiliated Hospital of Wenzhou Medical University. Written informed consent for participation was not required for this study in accordance with the national legislation and the institutional requirements.

## Authors Contributions

Conceptualization: S-QC, Y-FS, and Y-JX. Funding acquisition: S-QC, KW. Resources: S-QC, Y-FS, KW, SF, XC, H-MY, X-WL, L-PZ, JZ, YM. Investigation: Y-JX, KW, Y-TZ, SF, H-MY, Y-QC. Formal analysis: Y-JX, KW, Y-TZ, SF. Writing–original draft: Y-JX. Writing–review & editing: S-QC and Y-FS. All authors contributed to the article and approved the submitted version.

## Funding statement

This work was supported by the Clinical Research Plan of SHDC (No. SHDC2020CR1004A), the State Key Program of National Natural Science Foundation of China (No: 81730097), the National Natural Science Foundation of China (No: 82072618), the Science and Technology Commission Foundation of Shanghai Municipality (No: 19411967300).

## Conflict of Interest

The authors declare that the research was conducted in the absence of any commercial or financial relationships that could be construed as a potential conflict of interest.

## Publisher’s Note

All claims expressed in this article are solely those of the authors and do not necessarily represent those of their affiliated organizations, or those of the publisher, the editors and the reviewers. Any product that may be evaluated in this article, or claim that may be made by its manufacturer, is not guaranteed or endorsed by the publisher.

## Abbreviations

HCC, hepatocellular carcinoma; TACE, transarterial chemoembolization; SBRT, stereotactic body radiation therapy; OS, overall survival; CT, computed tomography; MRI, magnetic resonance imaging; JSH, Japan Society of Hepatology; PFS, progression-free survival; TRAEs, treatment-related adverse events; HR, hazard ratio; OR, odds ratio; ORR, objective response rate; DCR, disease control rate; DAMPs, damage-associated molecular patterns.
