# Supplementary material for: Effects of Stereotactic Body Radiation Therapy Plus PD-1 Inhibitors for Patients With Transarterial Chemoembolization Refractory
Source: Front Oncol. 2022 Mar 21;12:839605. doi: 10.3389/fonc.2022.839605 (PMC8978966; doi:10.3389/fonc.2022.839605)
Supplement: Supplementary file 1 [file Table_1.docx]

Supplementary Table 1. The PD-1 inhibitor of the study patients

| PD-1 inhibitor | Entire group  (n=76) | TACE-IO  (n=45) | SBRT-IO  (n=31) |
| --- | --- | --- | --- |
| Toripalimab | 55 (72.4) | 32 (71.1) | 23 (74.2) |
| Sintilimab | 21 (27.6) | 13 (28.9) | 8 (25.8) |
