# Supplementary material for: Effects of Stereotactic Body Radiation Therapy Plus PD-1 Inhibitors for Patients With Transarterial Chemoembolization Refractory
Source: Front Oncol. 2022 Mar 21;12:839605. doi: 10.3389/fonc.2022.839605 (PMC8978966; doi:10.3389/fonc.2022.839605)
Supplement: Supplementary file 2 [file Table_2.docx]

Supplementary Table 2. First site of progression and treatment on progression

|  | TACE-IO  (n=45) | SBRT-IO  (n=31) |
| --- | --- | --- |
| First site of progressive disease | 31 (68.9%) | 14 (45.2%) |
| Intrahepatic progression | 26 (57.8%) | 11 (35.5%) |
| In-field lesion | / | 9 (81.8%) |
| Out-field lesion | / | 2 (18.2%) |
| Extrahepatic progression | 3 (6.7%) | 2 (6.5%) |
| Intrahepatic progression and Extrahepatic progression | 2 (4.4%) | 1 (3.2%) |
| Treatment on progression | | |
| HAIC | 16 (35.6%) | 2 (6.5%) |
| MWA | 1 (2.2%) | 4 (12.9%) |
| Systemic therapy | 10 (22.2%) | 6 (19.4%) |
| Supportive care/refused treatment | 4 (8.9%) | 2 (6.5%) |

*TACE, transcatheter arterial chemoembolization; SBRT, stereotactic body radiation therapy; HAIC, hepatic artery infusion chemotherapy; MWA, microwave ablation.*
