# Supplementary material for: Effects of Stereotactic Body Radiation Therapy Plus PD-1 Inhibitors for Patients With Transarterial Chemoembolization Refractory
Source: Front Oncol. 2022 Mar 21;12:839605. doi: 10.3389/fonc.2022.839605 (PMC8978966; doi:10.3389/fonc.2022.839605)
Supplement: Supplementary file 3 [file Table_3.docx]

Supplementary Table 3. Treatment-related adverse events and Child-Pugh score progression

| Adverse Events | TACE-IO  (n=45) |  | SBRT-IO  (n=31) |  |
| --- | --- | --- | --- | --- |
|  | Any grade, n (%) | Grade 3/4. n (%) | Any grade, n (%) | Grade 3/4. n (%) |
| Decreased albumin | 17 (37.8) | 0 | 7 (22.6) | 0 |
| Decreased PLT | 20 (44.4) | 3 (6.7) | 15 (48.4) | 0 |
| Elevated AST | 17 (37.8) | 2 (4.4) | 8 (25.8) | 1 (3.2) |
| Elevated ALT | 13 (28.9) | 2 (4.4) | 7 (22.6) | 1 (3.2) |
| Decreased WBC | 10 (22.2) | 0 | 10 (32.3) | 0 |
| Elevated TB | 10 (22.2) | 0 | 8 (25.8) | 0 |
| Prolonged PT | 6 (13.3) | 0 | 4 (12.9) | 0 |
| Diarrhea | 6 (13.3) | 0 | 6 (19.4) | 0 |
| Fatigue | 13 (28.9) | 0 | 17 (54.8) | 0 |
| Hand-foot skin reaction | 9 (20.0) | 2 (4.4) | 7 (22.6) | 1 (3.2) |
| Decreased appetite | 4 (8.9) | 0 | 1 (3.2) | 0 |
| Infusion-related reaction | 11 (24.4) | 0 | 9 (29.0) | 0 |
| Progression of Child–Pugh score ≥2 | 10 (22.2) | | 2 (6.5) | |

*TACE, transcatheter arterial chemoembolization; SBRT, stereotactic body radiation therapy; PLT, platelet; AST,* *aspartate transaminase; ALT,* *alanine aminotransferase; WBC, white blood cell; TB, total bilirubin; PT,* *prothrombin time.*
